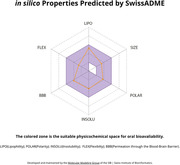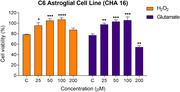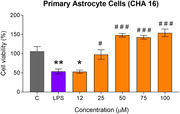# Chalcone derivate as a promising candidate for treating Alzheimer’s disease

**DOI:** 10.1002/alz.091121

**Published:** 2025-01-09

**Authors:** Murillo Orsatto Haas, Thaís C Ruaro, Grace Gosmann, Aline R. Zimmer

**Affiliations:** ^1^ Federal University of Rio Grande do Sul, Porto Alegre, Rio Grande do Sul Brazil; ^2^ Federal University of Rio Grande do Sul, Porto Alegre Brazil

## Abstract

**Background:**

Alzheimer’s disease (AD) is a progressive and multifactorial neurodegenerative disease that still has no cure. Different pathological processes contribute to the disease’s development, such as the presence of amyloid beta (Aβ) plaques, neurofibrillary tangles (NFTs), glutamatergic excitotoxicity, oxidative stress, and neuroinflammation^1^. Chalcones are polyphenolic compounds of natural origin with a wide range of biological activities^2^, and emerging studies have reported neurotrophic activity, anti‐inflammatory and antioxidant effects, and the inhibition of Aβ aggregation. In this study, we developed a series of innovative molecules based on chalcones and evaluated their cytotoxicity and neuroprotective effects *in silico* and *in vitro* experimental platforms.

**Methods:**

The MarvinSketch and SwissADME platforms were used to predict the drug‐like properties of the compounds and their ability to permeate the blood‐brain barrier (BBB). The *in vitro* assays of the 19 compounds were performed in C6 astroglial and VERO cell lines to assess their cytotoxicity. After that, their neuroprotective effects were evaluated against oxidative and glutamatergic insults in the C6 astroglial cell line. The 3 most promising compounds were tested in a primary culture of astrocytes from the cerebral cortex of a neonatal Wistar rat, and their ability to protect the cell from neuroinflammatory insult (LPS) was evaluated.

**Results:**

The *in silico* results indicated that all compounds presented satisfactory drug‐like properties and low or no cytotoxicity. Among tested compounds in *in vitro* assays, the most promising candidate was CHA 16. It showed a suitable oral bioavailability and a high potential to cross the BBB *in silico* assays, low cytotoxicity, and a neuroprotective effect against the oxidative and glutamatergic insults in C6 astroglial cells and against the inflammatory insult in primary astrocyte cells. In addition, CHA 16 displayed significant astro‐trophic effects in both cells tested.

**Conclusion:**

The compound CHA 16 protected the astrogial cells from oxidative, neuroinflammatory, and glutamatergic toxicity. Together with the high potential for oral use and passage through BBB, it is a promising candidate for clinical testing targeting AD treatment.

**References**:

1 Therriault, J. *et al. Trends in Molecular Medicine. Volume 28,9:726‐741*,

2 GOMES, M.N. et al. Chalcone Derivatives: Promising Starting Points for Drug Design. Molecules, 22:1210, 2017